# Co‐overexpression of the caloric restriction‐induced mitochondrial factors PGC‐1α and MIPEP upregulates *Phospho1* expression in adipocytes

**DOI:** 10.1002/2211-5463.70077

**Published:** 2025-06-26

**Authors:** Mamiko Ishimatsu, Kanari Taki, Asuka Hayami, Komachi Kato, Yuka Nozaki, Yuhei Mizunoe, Takumi Narita, Ryoichi Mori, Yoshikazu Higami, Masaki Kobayashi

**Affiliations:** ^1^ Laboratory of Molecular Pathology and Metabolic Disease, Faculty of Pharmaceutical Sciences Tokyo University of Science Noda Japan; ^2^ Department of Food and Nutrition Science, Graduate School of Humanities and Sciences Ochanomizu University Tokyo Japan; ^3^ Clinical Research Support Center Mie University Hospital Tsu Japan; ^4^ Department of Tissue Repair and Regenerative Medical Science, Atomic Bomb Disease Institute Nagasaki University Nagasaki Japan; ^5^ Leading Medical Research Core Unit, Graduate School of Biomedical Sciences Nagasaki University Nagasaki Japan; ^6^ Division of Cell Fate Regulation, Research Institute for Biomedical Sciences Tokyo University of Science Noda Japan; ^7^ The Institute for Human Life Science Ochanomizu University Tokyo Japan

**Keywords:** adipocytes, caloric restriction, MIPEP, mitochondria, PGC‐1α, PHOSPHO1

## Abstract

Peroxisome proliferator‐activated receptor gamma coactivator 1‐alpha (PGC‐1α) is a master transcriptional cofactor for mitochondrial biogenesis. Mitochondrial intermediate peptidase (MIPEP), a mitochondrial signal peptidase, plays an important role in the maturation and activation of mitochondrial proteins. Caloric restriction has lifespan‐extending effects that are reportedly exerted through induced expression of PGC‐1α and MIPEP in white adipose tissue. To evaluate how upregulation of PGC‐1α and MIPEP contributes to changes in the cellular characteristics of adipocytes, this study examined the mitochondrial function and differentiation of 3T3‐L1 preadipocytes with single overexpression (OE) or double OE of *Pgc‐1α* and *Mipep*. Compared with single‐OE cells, double‐OE cells exhibited no significant changes in oxygen consumption rate or mitochondrial morphology, but did show increased mitochondrial DNA levels. White adipocyte cell differentiation was suppressed in both *Pgc‐1α* single‐OE cells and double‐OE cells. Notably, double‐OE cells exhibited increased mRNA levels of phosphoethanolamine/phosphocholine phosphatase 1 (*Phospho1*), which plays a role in phospholipid metabolism and non‐canonical thermogenesis. *Phospho1* expression was also increased in white adipose tissue of mice under caloric restriction. In summary, the double OE of *Pgc‐1α* and *Mipep* induced *Phospho1* expression and suppressed adipocyte maturation, with little effect on mitochondrial function. This study provides new insights into the mitochondria‐related mechanism of caloric restriction in adipocytes.

## Graphical abstract

Caloric restriction that extends lifespan induces the expression of PGC‐1α and MIPEP in white adipose tissue. In this study, co‐overexpression of *Pgc‐1α* and *Mipep* upregulated the gene expression of PHOSPHO1. These findings provide new insights into mitochondria‐related mechanisms underlying the effects of caloric restriction in adipocytes.

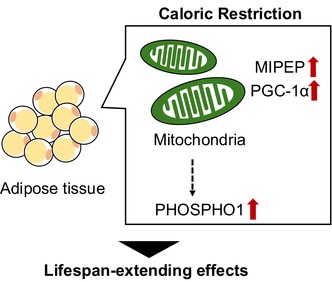

**Abbreviations**
BAT: brown adipose tissue
C/EBP: CCAAT enhancer binding protein
FCCP: carbonyl cyanide 4‐(trifluoromethoxy)phenylhydrazone
CKB: creatine kinase B
CR: caloric restriction
DOE: double overexpression
KLF: Kruppel‐like factor
MIPEP: mitochondrial intermediate peptidase
MtSPase: mitochondrial signal peptidase
NRF: nuclear respiratory factor
OCR: oxygen consumption rate
OE: overexpression
PGC: peroxisome proliferator‐activated receptor gamma coactivator
PHOSPHO1: phosphoethanolamine/phosphocholine phosphatase 1
PLIN1: perilipin 1
PPAR: peroxisome proliferator‐activated receptor
PRDM16: PR domain‐containing 16
RT‐PCR: quantitative real‐time PCR
SIRT3: sirtuin 3
TFAM: transcription factor A mitochondria
UCP1: uncoupling protein
WAT: white adipose tissue

Caloric restriction (CR) is a robust and reproducible manipulation that prevents many age‐related pathophysiologic events and extends both median and maximum lifespan [1, 2]. These effects of CR have been observed in various species, including yeasts, worms, rodents, and even nonhuman primates, suggesting that CR could also be beneficial for humans [3] and inspiring research on the mechanisms underlying the therapeutic effects of CR. Previous studies have demonstrated that CR enhances mitochondrial biogenesis, specifically by increasing the abundance of mitochondrial DNA (mtDNA) and the expression of mitochondrial genes and proteins [4, 5, 6, 7, 8]. In skeletal muscle, however, CR reportedly improves mitochondrial function without enhancing mitochondrial biogenesis [9]. These findings support a close relationship between mitochondria and CR effects, but the precise mechanism remains controversial.

White adipose tissue (WAT), a major organ that stores energy in the form of triglyceride (TG), is now considered an endocrine tissue with the ability to secrete adipokines, including *Adipoq*‐encoded adiponectin and leptin [10]. WAT mainly comprises white adipocytes with unilocular TG lipid droplets. Various molecular mechanisms involved in the differentiation of preadipocytes into mature adipocytes have been previously identified using preadipocyte cell lines. For example, in the early stage of adipocyte differentiation, the expression of Kruppel‐like factor 5 (KLF5), CCAAT enhancer binding protein (C/EBP)β, and C/EBPδ is induced, while that of KLF2 and KLF3 is suppressed. In the late stage, the expression of C/EBPα and peroxisome proliferator‐activated receptor (PPAR)γ is induced, followed by upregulation of mature adipocyte markers, including adiponectin and perilipin 1 (PLIN1), a major lipid droplet‐coating protein [11, 12]. By contrast, brown adipose tissue (BAT) contains multilocular lipid droplets and plays a central role in energy dissipation through heat production. Prior studies have demonstrated the cold exposure‐ or adrenergic activation‐induced alteration of white adipocytes into brown‐like adipocytes with the capacity for thermogenesis, called “beige fat” [13, 14]. Uncoupling protein 1 (UCP1), a representative marker of BAT and beige fat, uncouples aerobic respiration by disturbing the inter‐membrane proton‐motive force, thereby producing heat instead of ATP [15]. Meanwhile, UCP1‐independent mechanisms in thermogenesis have also been reported, including futile creatine cycling and Ca^2+^ cycling [16, 17, 18]. PR domain‐containing 16 (PRDM16) is accepted as a pivotal transcriptional co‐regulator of brown adipogenesis [19].

Phosphoethanolamine/phosphocholine phosphatase 1 (PHOSPHO1), which hydrolyzes phosphocholine and phosphoethanolamine into choline and ethanolamine, respectively, regulates the biomineralization of bone and other hard tissues [20]. Interestingly, Kazak et al. found that PHOSPHO1 is induced in beige fat of *Ucp1*‐deficient mice [16]. Jiang et al. further reported abundant expression of *Phospho1* in BAT and accumulation of *Phospho1* transcripts in brown adipocytes undergoing differentiation [21]. These studies suggest that PHOSPHO1 plays a critical role in brown adipogenesis or differentiation of cells into beige adipocytes.

Peroxisome proliferator‐activated receptor gamma coactivator (PGC)‐1α is a master transcriptional cofactor for mitochondrial biogenesis that regulates the expression of various mitochondria‐related genes and the replication of mitochondrial DNA (mtDNA) [22, 23]. Representative downstream target genes of PGC‐1α include mitochondrial transcription factor A (TFAM), which is required for mtDNA replication and transcription, and sirtuin 3 (SIRT3), a mitochondria‐localized deacetylase that modulates the acetylation levels of mitochondrial enzymes [24, 25, 26]. We previously demonstrated that CR increases the transcription of *Pgc‐1α* in WAT in a manner dependent on sterol regulatory element binding protein 1c (SREBP‐1c), a master transcriptional factor of fatty acid biosynthesis [27]. The involvement of PGC‐1α in CR‐induced mitochondrial biogenesis was established by other researchers, who showed that CR‐induced upregulation of mitochondrial‐related genes and mtDNA levels was abolished in WAT of mice lacking both PGC‐1α and PGC‐1β [28]. PGC‐1α is also a transcriptional regulator of thermogenesis in BAT [29].

In addition to PGC‐1α, we identified mitochondrial intermediate peptidase (MIPEP) as an SREBP‐1c‐dependent CR‐induced mitochondrial factor in WAT [30, 31]. MIPEP is a member of the mitochondrial signal peptidase (MtSPase) family, which plays an important role in the maturation of mitochondrial proteins transported from the cytosol to mitochondria [32]. Mechanistically, MIPEP cleaves mitochondrial proteins that have already been processed by another MtSPase (mitochondrial processing peptidase), thus performing the second of two successive cleavage steps leading to the maturation and activation of these proteins [33, 34]. Previously, we showed that MIPEP contributes to the maturation of SIRT3, while CR increases its mature forms in WAT [30]. These findings raise the possibility that the actions of PGC‐1α and MIPEP in CR‐related qualitative changes of WAT are coordinated via the control of mitochondrial biogenesis or function.

In this study, to evaluate the contribution of upregulation of PGC‐1α and MIPEP on the cellular characteristics of adipocytes, we examined mitochondrial function and adipogenesis in 3T3‐L1 adipocytes with double overexpression (DOE) of *Pgc‐1α* and *Mipep*. Compared with single overexpression (OE) of *Pgc‐1α* or *Mipep*, DOE cells showed elevated levels of mtDNA, but no significant differences in other mitochondrial phenotypes. White adipocyte differentiation was suppressed in cells with DOE or *Pgc‐1α* single OE. Only DOE cells exhibited increased expression levels of *Phospho1*, which was confirmed in the WAT of CR mice. Taken together, our findings showed that the DOE of *Pgc‐1α* and *Mipep* suppressed adipocyte maturation and transcriptionally induced *Phospho1*, without directly affecting mitochondrial function.

## Materials and methods

### Animals and diet

The animal experiments were approved by the Ethical Review Committee for Animal Experimentation at the Tokyo University of Science (Y16049 and Y17051). Male mice (C57BL/6) and male rats (Wistar) were maintained at the Laboratory Animal Center, Faculty of Pharmaceutical Sciences, Tokyo University of Science as follows: specific pathogen‐free conditions, 23°C temperature, 12‐h light/dark cycle, water, and a CRF‐1 diet (Oriental Yeast, Tokyo, Japan). Starting at 12 weeks of age, mice and rats were divided into two feeding groups: *ad libitum* (AL) and CR (70% of AL energy intake). At 9 months of age, each group of mice and rats was euthanized under isoflurane anesthesia (Wako, Osaka, Japan) for the collection of epididymal WAT (mice: AL, *n* = 5; CR, *n* = 6; rats: AL, *n* = 5; CR, *n* = 6). The WAT samples were immediately diced, frozen in liquid nitrogen, and stored at −80 °C. Body and tissue weights of the mice and rats are shown in Table 1.

**Table 1:** Body and tissue weights of mice and rats analyzed in this study.

Body and tissue weight		
Mouse	AL	CR
Body weight (g)	32.22 ± 2.51	26.12 ± 2.99
eWAT (g)	0.45 ± 0.14	0.14 ± 0.06

Rat	AL	CR
Body weight (g)	531.68 ± 33.51	396.49 ± 2.85
eWAT (g)	7.78 ± 1.64	2.85 ± 0.40

### Cell culture and differentiation

3T3‐L1 preadipocytes obtained from the Japanese Collection of Research Bioresources were cultured in low‐glucose DMEM (Wako) supplemented with 10% fetal bovine serum (Thermo Fisher Scientific, Waltham, MA, USA) and 1% penicillin/streptomycin (Sigma‐Aldrich, St. Louis, MI, USA). For differentiation into mature adipocytes, 3T3‐L1 cells were seeded and cultured at a cell density of 1.5 × 10^4^ cells·cm^−2^. When 100% confluence was reached after 2 days, the cells were cultured in normal medium containing 500 μm 3‐isobutyl‐1‐methylxanthine (Sigma‐Aldrich) and 1 μm dexamethasone (Sigma‐Aldrich) for 2 days. Differentiation into mature adipocytes was achieved by culturing in normal medium containing 10 μg·mL^−1^ insulin (Sigma‐Aldrich) and 50 nm tri‐iodothyronine (Wako), which was replaced every 2 days thereafter. After 2 days of differentiation, 3T3‐L1 cells were treated with 1 or 5 μm etomoxir (Sigma‐Aldrich), a CPT‐1 inhibitor, to inhibit β‐oxidation. This procedure was repeated at 2‐day intervals for 8 days, and the cells were collected at 10 days of differentiation.

### Plasmid construction

The coding region of *Mipep* was amplified from mouse cDNA using the following primers: forward 5′‐GTA CGG GAA TTC GCC ACC ATG CTG CTG GCG GCC GG‐3′ and reverse 5′‐CGG CCG CTC GAG CTA TTT AGA ATC CAG GAA GAA CG‐3′. PCR was performed using PrimeSTAR HS polymerase (Takara, Tokyo, Japan), in accordance with the manufacturer's protocol. This amplicon was digested with *Eco*RI and *Xho*I and inserted into the pMXs‐AMNN‐Puro plasmid [27], generating pMXs‐AMNN‐*Mipep*‐Puro. *Pgc1α* cDNA obtained from *Eco*RV and *Pme*I‐digested pcDNA‐f:PGC1 (Cat# 1026; Addgene, Cambridge, MA, USA) was cloned into *Eco*RI‐digested, Klenow fragment‐blunted pMXs‐Neo (Nippon Gene, Tokyo, Japan) and dephosphorylated with alkaline phosphatase (Takara) to generate pMXs‐*Pgc1α*‐Neo.

### Retroviral vector preparation and establishment of OE cells

3T3‐L1 cells with single OE or DOE of *Mipep* and *Pgc‐1α* were generated using retroviral vectors, as previously reported [25]. The pMXs‐AMNN‐*Mipep*‐Puro plasmid was transfected into the Plat‐E retroviral packaging cell line (kindly provided by T. Kitamura) using the calcium phosphate method. After 2 days, the supernatant from each virus‐containing culture was harvested and passed through a 0.22‐μm filter (Millipore, Billerica, MA, USA). To establish *Mipep* single‐OE cells, undifferentiated 3T3‐L1 cells were incubated in virus‐containing medium for 24 h, followed by selection with 2 μg·mL^−1^ puromycin (Wako) for 5–7 days. The virus engineered for *Pgc‐1α* single OE was prepared by the same method using the pMXs‐*Pgc‐1α*‐Neo plasmid. To establish *Mipep* and *Pgc‐1α* DOE cells, *Mipep* OE cells were infected with the *Pgc‐1α* OE virus for 24 h, followed by selection with 1 mg·mL^−1^ G418 (Cayman Chemical, Ann Arbor, MI, USA) for 5–7 days. Selected *Mipep* and *Pgc‐1α* DOE cells were cultured and maintained in medium containing 0.4 μg·mL^−1^ puromycin and 200 μg·mL^−1^ G418.

### Protein extraction and immunoblotting

Cells were lysed with lysis buffer (50 mm Tris/HCL, pH 6.8, 2% sodium dodecyl sulfate, 5% glycerol), boiled for 5 min, and sonicated. Protein concentrations within the soluble fractions were determined using a Pierce BCA Protein Assay Kit (Thermo Fisher Scientific), in accordance with the manufacturer's protocol. Extracted protein samples were standardized by the addition of lysis buffer containing 2‐mercaptoethanol and bromophenol blue (final concentrations of 5% and 0.025%, respectively) and then boiled for 5 min. Fifteen micrograms of each protein sample was subjected to SDS/PAGE on 10% acrylamide gels and then transferred to nitrocellulose membranes. Membranes were blocked with 2.5% skim milk and 0.25% bovine serum albumin in Tris‐buffered saline (50 mm Tris, pH 7.4 and 150 mm NaCl) containing 0.1% Tween 20 for 60 min at room temperature, and then probed with primary antibodies against the following proteins overnight at 4°C: MIPEP (custom antibody produced by Eurofins Genomics, Luxembourg City, Luxembourg), PGC‐1α (Cat# AB3242; Millipore), and SIRT3 (Cat# 5490; Cell Signaling, Danvers, MA, USA). The membranes were then incubated for 1 h at room temperature with the appropriate secondary antibodies: horseradish peroxidase‐conjugated F(ab’)2 fragment of goat anti‐rabbit IgG (Jackson ImmunoResearch, West Grove, PA, USA). Antibody‐bound proteins were visualized using ImmunoStar LD reagent (Wako) and ChemiDoc™ Touch and data were analyzed using Image Lab software (Bio‐Rad, Hercules, CA, USA). Specific signal intensities were normalized to those of Ponceau S (Beacle, Inc., Kyoto, Japan) staining or LAMINB1.

### Quantitative real‐time PCR (RT‐PCR)

Total RNA was extracted from cells and WAT using ISOGEN II (Nippon Gene), and reverse transcribed using ReverTra Ace® qPCR RT Master Mix (Toyobo, Osaka, Japan), in accordance with the manufacturers' protocols. RT‐PCR was performed using the CFX Connect™ Real‐Time System (Bio‐Rad) and Thunderbird SYBR qPCR mix (Toyobo). Target gene expression data were normalized to the mRNA expression levels of ribosomal protein S18 (*Rps18*). Primer sequences are shown in Table 2.

**Table 2:** The sequences of primers used for RT‐PCR in this study.

Target gene	Forward	Reverse
*Rps18*	5′‐TGC GAG TAC TCA ACA CCA ACA T‐3′	5′‐CTT TCC TCA ACA CCA CAT GAG C‐3′
*Pgc‐1α*	5′‐AGA CGG ATT GCC CTC ATT TG‐3′	5′‐CAG GGT TTG TTC TGA TCC TGT G‐3′
*Mipep*	5′‐CAA AGG AGA GGT GTG GTG TAA TG‐3′	5′‐GGA AGA TTC AGC ATG AGA ACG AC‐3′
*Sirt3*	5′‐CGT TGT GAA ACC CGA CAT TG‐3′	5′‐TCC CCT AGC TGG ACC ACA TC‐3′
*Tfam*	5′‐CGG CTC AGG GAA AAT TGA AG‐3′	5′‐TCC AAC TTC AGC CAT CTG CTC‐3′
*Pparγ*	5′‐CAC AAT GCC ATC AGG TTT GG‐3′	5′‐GCG GGA AGG ACT TTA TGT ATG AG‐3′
*PeriA*	5′‐TGG GAA GCA TCG AGA AGG TG‐3′	5′‐ATG GTG TGT CGA GAA AGA GTG TTG‐3′
*Adipoq*	5′‐TGC CGA AGA TGA CGT TAC TAC AAC‐3′	5′‐CTT CAG CTC CTG TCA TTC CAA C‐3′
*Cebpa*	5′‐GCC TTC AAC GAC GAG TTC C‐3′	5′‐CCC GGG TAG TCA AAG TCA CC‐3′
*Cebpb*	5′‐AAG ATG TTC CTG CGG GGT TG‐3′	5′‐CAC TTT AAT GCT CGA AAC GGA AAA G‐3′
*Cebpd*	5′‐GTT CAT TCT CTC CCG CAC AC‐3′	5′‐GAA ACG CGT CCA TCT CCT TAC‐3′
*Klf2*	5′‐ACC AAG AGC TCG CAC CTA AA‐3′	5′‐TCC TTC CCA GTT GCA ATG AT‐3′
*Klf3*	5′‐TAC AGG AGA AAA GCC GTA CAA ATG‐3′	5′‐TCA TCA GAC CGA GCG AAC TTC‐3′
*Klf5*	5′‐CCG GAG ACG ATC TGA AAC AC‐3′	5′‐GGA GCT GAG GGG TCA GAT ACT T‐3′
*Phospho1 (mouse)*	5′‐AAG CAC ATC ATC CAC AGT CCC‐3′	5′‐TGT TGG TCT CCA GCT GTC AT‐3′
*Phospho1 (rat)*	5′‐TGA GCC GGA GCT CAG AAA TC‐3′	5′‐CCA CCT TTA GAA ACT TGG TTG GTC‐3′
*Ckb*	5′‐AGT TCC CTG ATC TGA GCA GC‐3′	5′‐GAA TGG CGT CGT CCA AAG TAA‐3′

### DNA extraction and measurement of mtDNA content

The evaluation of mtDNA content was performed according to a previously reported method [35, 36]. Total DNA was extracted from cell lysates via digestion with 100 μg·μL^−1^ proteinase K in a lysis buffer comprising 150 mm NaCl, 10 mm Tris/HCl (pH 8.0), 10 mm EDTA, 0.1%SDS. After an overnight incubation at 55 °C, an equal volume of TE saturated phenol was added, and samples were rotated for one hour. After centrifugation, an equal volume of PCI (phenol/chloroform/isoamyl alcohol) was added to the gained aqueous phase, and the DNA fraction were harvested after continuous rotation for one hour and centrifugation. To eliminate RNA contamination, the DNA fraction was treated with a 0.02‐volume RNase A solution (Wako, Osaka, Japan) at 37 °C for one hour. The phenol and PCI extraction steps as mentioned above were repeated once. The DNA was then precipitated by adding 0.1 volume of 3 m sodium acetate and two volumes of ethanol. The DNA pellet was washed twice with 70% ethanol, air‐dried, and dissolved in TE buffer. The extracted DNA was then subjected to quantitative PCR as follows. The mtDNA‐encoded cytochrome c oxidase subunit II (*CoxII*) gene was amplified with the primers forward: 5′‐CCA TCC CAG GCC GAC TAA‐3′ and reverse: 5′‐AAT TTC AGA GCA TTG GCC ATA GA‐3′. The genomic hypoxanthine‐guanine phosphoribosyl transferase (*Hprt*) gene was amplified using the primers forward: 5′‐TGG GAG GCC ATC ACA TTG T‐3′ and reverse: 5′‐TCC AGC AGG TCA GCA AAG AA‐3′. Quantitative PCR was performed using a CFX Connect™ Real Time System and a Thunderbird SYBR qPCR mix in accordance with the manufacturer's protocols. Relative mtDNA content is represented as the amplification ratio of *CoxII* to *Hprt*.

### Oxygen consumption rate (OCR) measurement

The OCR of cells in Agilent Seahorse XF DMEM medium, pH 7.4, containing 2 mm glutamine, 10 mm D‐glucose, and 1 mm pyruvate was measured using a Seahorse XF analyzer (Agilent Technologies, Santa Clara, CA, USA). During the measurement, modulators of cellular respiration were added in the following order: 1 μm oligomycin (Cayman Chemical), 0.25 μm carbonyl cyanide 4‐(trifluoromethoxy)phenylhydrazone (FCCP; Cayman Chemical), 0.5 μm rotenone (Sigma‐Aldrich), and 0.5 μm antimycin (Sigma‐Aldrich).

### Oil red O staining of 3T3‐L1 cells

Differentiated 3T3‐L1 cells were fixed with 10% formalin solution (neutral buffered) and then stained with oil red O (Sigma‐Aldrich) staining solution (1.8 mg·mL^−1^ in 60% isopropanol) for 20 min at room temperature. After washing with 60% isopropanol and H_2_O, the stained cells were visualized under a BZ‐9000 light microscope (Keyence, Osaka, Japan). The stained cells were then incubated in isopropanol for 1 h at room temperature to dissolve the staining solution. The absorbance of oil red O in the lysate was then measured at 490 nm using an ARVO MX/Light Wallac 1420 Multilabel/Luminescence Counter (Perkin Elmer, Waltham, MA, USA).

### Transmission electron microscopy (TEM)

Cells were differentiated into adipocytes and then fixed with 2.5% glutaraldehyde in phosphate buffer. TEM was performed as previously described [37].

### Statistical analysis

Values are expressed as means ± standard deviation. Differences between multiple groups were analyzed using the Tukey–Kramer test, while those between two groups were analyzed using Student's t‐test. Differences with *P* values <0.05 were considered statistically significant.

## Results

### DOE of *Pgc‐1α* and *Mipep* does not markedly affect mitochondrial function or morphology

**Fig. 1:**
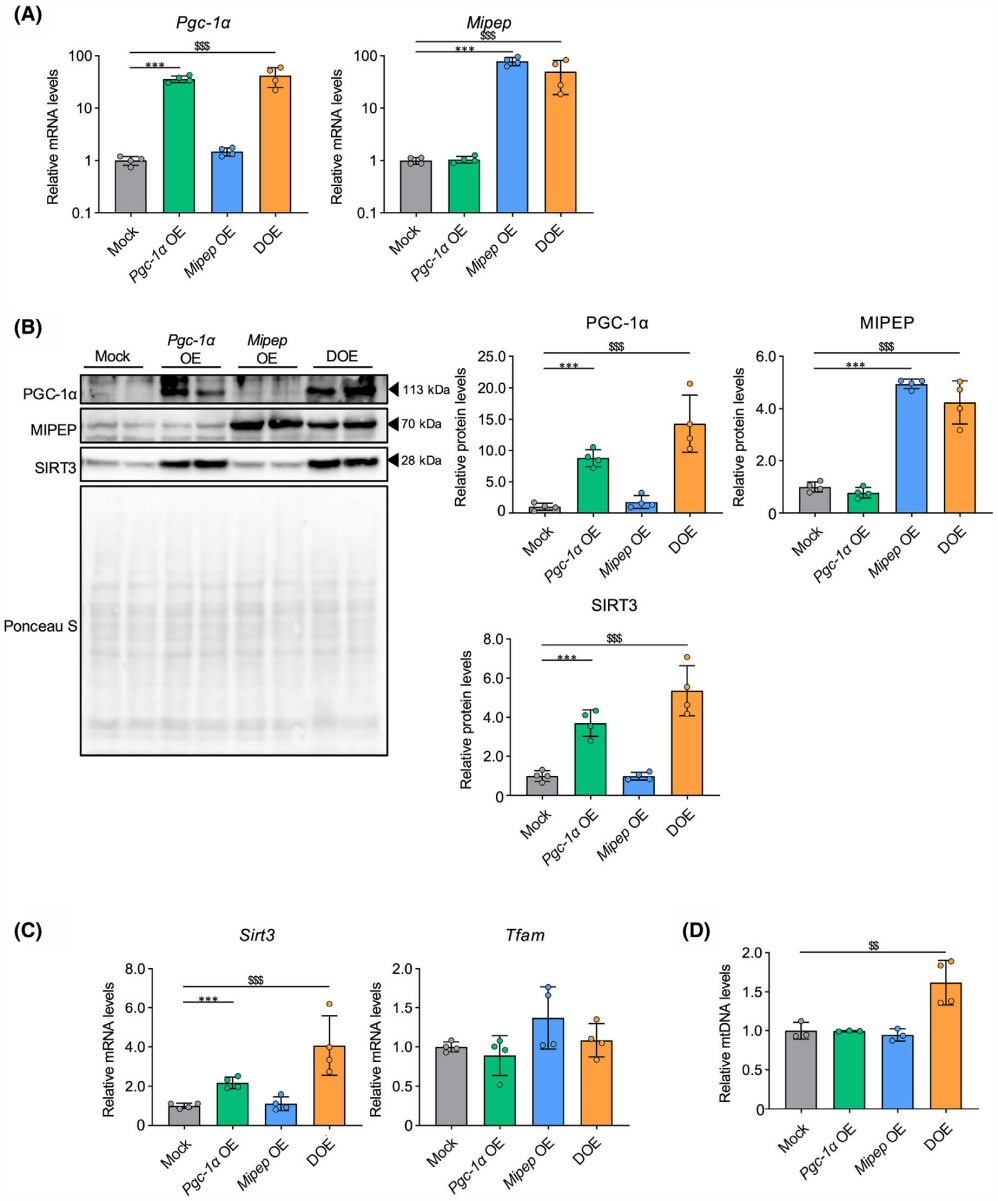
Levels of mitochondrial factors in 3T3‐L1 cells with single overexpression (OE) or double OE (DOE) of *Pgc‐1α* and *Mipep*. (A) Quantitative real‐time PCR (RT‐PCR) analysis of mRNA expression levels of *Pgc‐1α* and *Mipep* in 3T3L1 cells with mock infection (Mock), single OE, or DOE of *Pgc‐1α* and *Mipep* (*n* = 4 per group). (B) Representative immunoblotting images (left) and quantification of relative levels (right) of PGC‐1α, MIPEP, and SIRT3 proteins in the four groups (*n* = 4 per group). (C) RT‐PCR analysis of mRNA levels of *Sirt3* and *Tfam* (*n* = 4 per group). (D) Mitochondrial DNA (mtDNA) content calculated as the relative ratio of mtDNA gene *CoxII* to nuclear gene *Hprt* (*n* = 4 per group). RT‐PCR data were normalized to *Rps18* expression levels. Ponceau S staining was used as a loading control for immunoblotting. Values expressed as means ± standard deviation. Differences were statistically analyzed using the Tukey–Kramer test; *P < 0.05, **P < 0.01, ***P < 0.005 for Mock vs. *Pgc‐1α* OE groups; ^$$^ *P* < 0.01, ^$$$^ *P* < 0.005 for Mock vs. DOE groups.

**Fig. 2:**
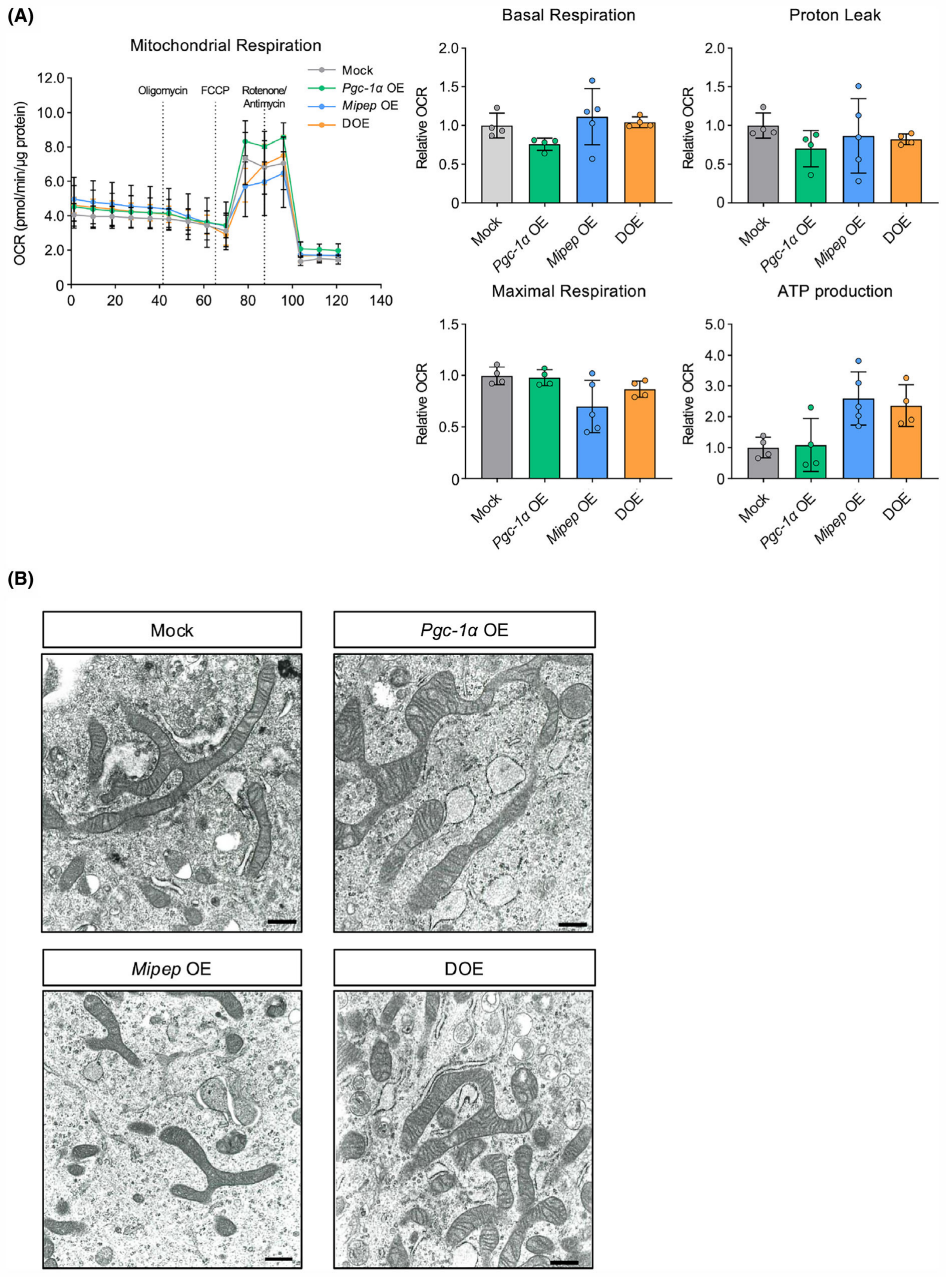
Evaluation of mitochondrial function and morphology in 3T3‐L1 cells with single overexpression (OE) or double OE (DOE) of *Pgc‐1α* and *Mipep*. (A) Oxygen consumption rate (OCR) measured after sequential addition of oligomycin, carbonyl cyanide 4‐(trifluoromethoxy)phenylhydrazone (FCCP), and rotenone/antimycin A in 3T3L1 cells with mock infection (Mock), single OE, or DOE of *Pgc‐1α* and *Mipep* (*n* = 4 per group). Graphs at the right represent the mitochondrial respiration parameters calculated from the OCR data. Values expressed as means ± standard deviation. (B) Representative transmission electron microscopy images. Scale bars = 0.5 μm.

Single OE and DOE of *Pgc‐1α* and *Mipep* were confirmed at the mRNA and protein levels in each stable OE cell line (Fig. 1A,B). We also observed that, compared with the findings in mock‐infected cells, SIRT3 expression was increased at the mRNA and protein levels in *Pgc‐1α* OE cells and DOE cells (Fig. 1B,C), whereas *Tfam* expression was unchanged (Fig. 1C). Additionally, mtDNA content, a representative marker of mitochondrial biogenesis, was increased in DOE cells but not in single‐OE cells (Fig. 1D). To assess mitochondrial function, we examined the OCR, which did not change in any of the stable OE cell lines (Fig. 2A). Using TEM images of the stable OE cell lines, we also evaluated mitochondrial morphology, which showed no changes in any of the stable OE cell lines (Fig. 2B).

### *Pgc‐1α* and *Mipep* DOE and *Pgc‐1α* OE suppress adipocyte maturation

**Fig. 3:**
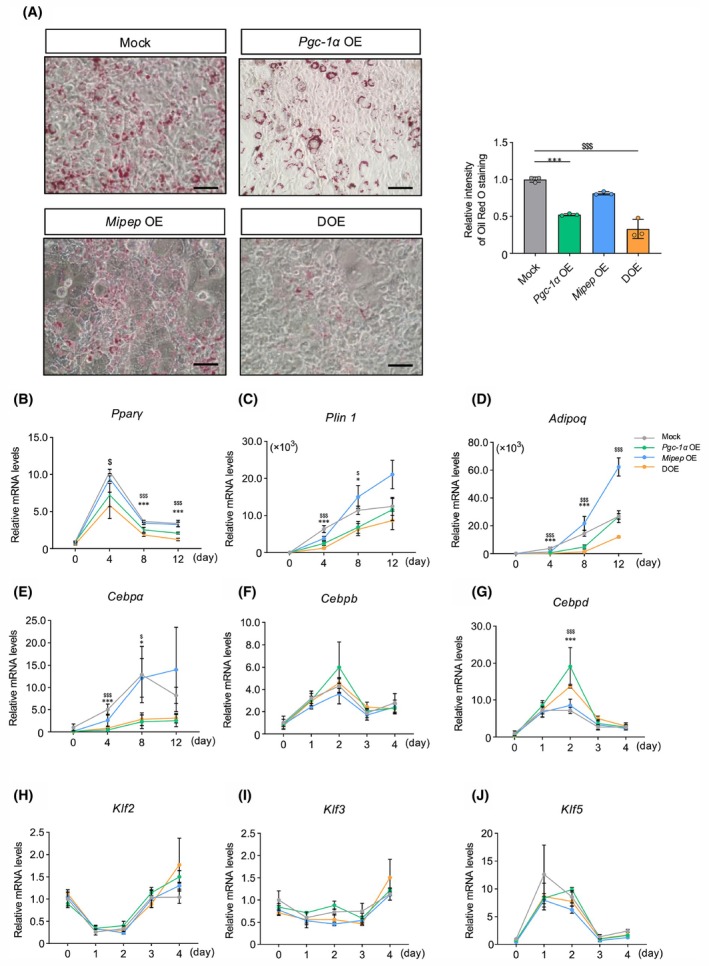
Evaluation of adipocyte maturation under single overexpression (OE) or double OE (DOE) of *Pgc‐1α* and *Mipep*. (A) Representative images (left) and quantitation of the relative intensity (right) of oil red O staining of lipids (left) in 3T3L1 cells with mock infection (Mock), single OE, or DOE of *Pgc‐1α* and *Mipep* (*n* = 3 per group). Scale bars = 100 μm. (B–D) Quantitative real‐time PCR (RT‐PCR) analysis of mRNA expression levels of the following adipocyte maturation marker genes at 0, 4, 8, and 12 days post‐induction of adipocyte differentiation: *Pparγ* (B), *Plin 1* (C), *Adipoq* (D), *Cebpa* (E) (*n* = 3 per group). (E–J) RT‐PCR analysis of mRNA levels of the following early‐stage adipocyte differentiation marker genes at 0, 1, 2, 3, and 4 days post‐induction of adipocyte differentiation: *Cebpb* (F), *Cebpd* (G), *Klf2* (H), *Klf3* (I), and *Klf5* (J) (*n* = 3 per group). RT‐PCR data were normalized to *Rps18* expression levels. Values expressed as means ± standard deviation. Differences were statistically analyzed using the Tukey–Kramer test; *P < 0.05, **P < 0.01, ***P < 0.005 for Mock vs. *Pgc‐1α* OE groups; ^$^ *P* < 0.05, ^$$^ *P* < 0.01, ^$$$^ *P* < 0.005 for Mock vs. DOE groups.

To examine the amount of lipid droplets in stable OE cell lines, we performed oil Red O staining. The results showed fewer lipid droplets in *Pgc‐1α* OE and DOE cells compared with the findings in mock‐infected cells (Fig. 3A). Because lipid accumulation in differentiated 3T3‐L1 cells is generally believed to be dependent on adipogenesis, we examined the sequential changes of adipocyte differentiation marker genes after inducing differentiation in stable OE cell lines. Among the markers involved in the late stage of adipocyte differentiation, the post‐induction levels of *Pparγ*, *Plin 1*, *Adipoq*, and *Cebpa* were decreased in both *Pgc‐1α* OE and DOE cells (Fig. 3B–E). By contrast, except for *Cebpd*, the levels of early‐stage adipocyte differentiation markers *Cebpb*, *Klf2*, *Klf3*, and *Klf5* did not significantly change in any of the stable OE cell lines (Fig. 3F–J). We also observed that treatment with etomoxir, an inhibitor of fatty acid oxidation, failed to restore the normal amount of lipid droplets in DOE cells, supporting the notion that the lipid decrease was a result of suppressed adipocyte maturation rather than activated fatty acid oxidation (Fig. S1). These results suggested that *Pgc‐1α* OE and *Pgc‐1α* and *Mipep* DOE suppress the late stage of adipocyte differentiation, also known as adipocyte maturation.

### *Pgc‐1α* and *Mipep* DOE and CR upregulate *Phospho1* levels in adipocytes and WAT

**Fig. 4:**
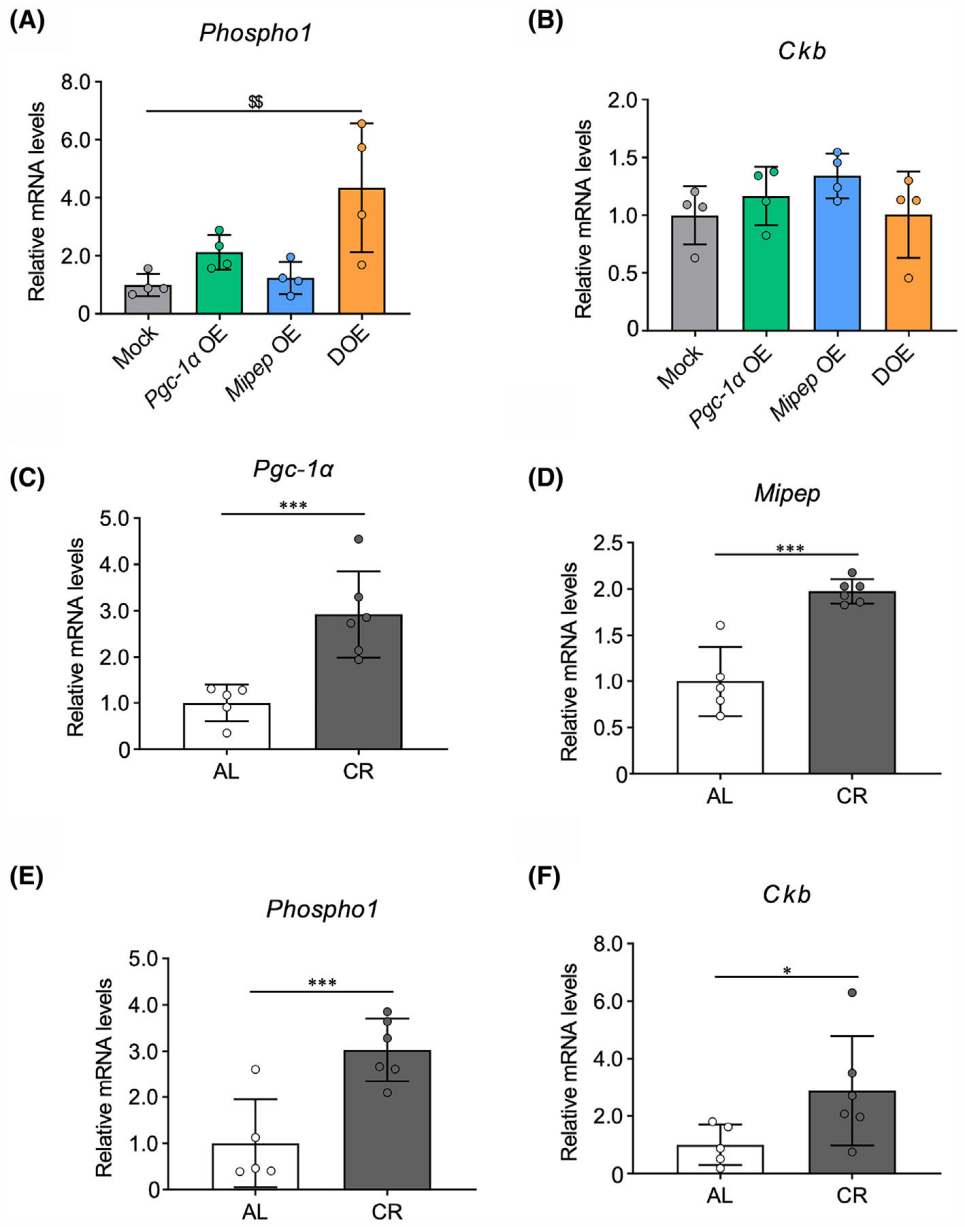
Creatine cycling‐related gene expression in 3T3‐L1 cells with *Pgc‐1α* and *Mipep* single overexpression (OE) and double OE (DOE), and white adipose tissue (WAT) of mice under caloric restriction (CR). (A, B) Quantitative real‐time PCR (RT‐PCR) analysis of mRNA expression levels of *Phospho1* (A) and *Ckb* (B) in in 3T3L1 cells with mock infection (Mock), single OE, or DOE of *Pgc‐1α* and *Mipep* (*n* = 4 per group). (C–F) RT‐PCR analysis of mRNA expression of *Pgc‐1α* (C), *Mipep* (D), *Phospho1* (E), and *Ckb* (F) in the WAT of two feeding groups of mice: *ad libitum* (AL; *n* = 5) and CR (*n* = 6). RT‐PCR data were normalized to *Rps18* expression levels. Values are means ± SD. Differences were statistically analyzed using the Tukey–Kramer test (A, B) or Student's *t*‐test (C–F); *P < 0.05, ***P < 0.005 for Mock vs *Pgc‐1α* OE or AL vs. CR groups; ^$$^ *P* < 0.01 for Mock vs. DOE groups.

Considering that PGC‐1α reportedly plays important roles in thermogenic capacity in brown adipocytes [38], we examined thermogenesis‐related genes in our OE cell lines. Expression of two representative thermogenesis marker genes*—Ucp1* and *Prdm16*—was not detected in any of the OE cell lines (data not shown), consistent with a report that these genes were not induced in 3T3‐L1 cells differentiated using the standard protocol [39, 40]. Therefore, we sought to examine gene expression in a UCP1‐independent thermogenesis pathway, namely the futile creatine cycle, in our OE cells. The results showed that the expression of *Phospho1*, a creatine cycling‐related gene, was increased in DOE cells, but not in either single‐OE cell line, whereas the expression of another creatine cycling‐related gene, *Ckb*, did not significantly change compared with that in mock‐infected cells (Fig. 4A,B).

Next, we analyzed the expression of *Phospho1* and *Ckb* in WAT of CR mice that were confirmed to exhibit increased levels of *Pgc‐1α* and *Mipep* compared with AL control mice (Fig. 4C,D). The results showed that CR significantly upregulated the levels of *Phospho1* and *Ckb* (Fig. 4E,F). Additionally, CR upregulated the levels of *Phospho1* in WAT of rats (Fig. S2). These findings suggested that DOE of *Pgc‐1α* and *Mipep* contributes to creatine cycling‐related thermogenesis in adipocytes via induced *Phospho1* expression.

## Discussion

The expression of mitochondrial‐related factors PGC‐1α and MIPEP in WAT is reportedly induced by CR [27, 28, 30]. In this study, we examined mitochondrial biogenesis, mitochondrial function, and adipogenesis in *Pgc‐1α* and *Mipep* DOE cells. *Pgc‐1α* and *Mipep* DOE promoted the expression of *Phospho1* and suppressed adipocyte maturation, with little effect on mitochondrial function.

PGC‐1α reportedly increases the transcription of several mitochondrial‐related genes, including *SIRT3* and *TFAM* [24, 25, 26]. While we observed an increase in the expression of SIRT3 in *Pgc‐1α* OE and DOE cells, unexpectedly, the expression of *Tfam* did not change (Fig. 1B,C). PGC‐1α‐induced promotion of downstream target genes is known to be mediated by the activation of nuclear transcription factors, including nuclear respiratory factors (NRFs) and estrogen‐related receptor α (ERRα). Specifically, PGC‐1α increases the gene expression of *Sirt3* via ERRα and that of *Tfam* via NRFs [24, 25]. The variation in transcriptional response of PGC‐1α target genes in *Pgc‐1α* OE and DOE cells might be attributable to these mechanistic differences.

mtDNA levels increased in DOE cells but not in single‐OE cells, although the expression of *Tfam* did not change (Fig. 1D), suggesting that PGC‐1α and MIPEP may co‐regulate mtDNA replication without an increase in the abundance of TFAM. In a recent study reporting that TFAM was a substrate of SIRT3, the authors also showed that OE of *Sirt3* activated TFAM by inducing its deacetylation at K154, which in turn enhanced mitochondrial biogenesis [41]. We previously demonstrated that MIPEP orchestrates the maturation and activation of SIRT3 [30]. Despite a lack of direct evidence, our current results and previous findings suggest that the *Pgc‐1α* and *Mipep* DOE‐induced expression and maturation of SIRT3 reinforce the activity of TFAM rather than its abundance, eventually increasing mtDNA levels in DOE cells. However, because DOE cells exhibited no significant functional or morphological changes in mitochondria (Fig. 2), we propose that the increase in mtDNA levels may reflect enhanced mitochondrial biogenesis, albeit at a level insufficient to cause obvious changes in mitochondria.

We found that *Pgc‐1α* OE and DOE adipocytes exhibited reductions in the amount of lipid droplets and the expression levels of mature adipocyte marker genes *Pparγ*, *Plin 1*, and *Adipoq*, whereas the expression levels of marker genes involved in the early stage of adipocyte differentiation barely changed (Fig. 3). A prior study demonstrated that induction of PGC‐1α attenuated the differentiation of skeletal stem cells into adipocytes [42], suggesting that PGC‐1α negatively regulates white adipocyte differentiation through an unknown mechanism. However, other studies have shown that CR elevates the expression levels of adipogenic differentiation markers in WAT [43, 44]. Taken together, it is conceivable that CR‐induced PGC‐1α may not be a major contributor to the adipogenic capacity or adipocyte maturation of WAT.

Interestingly, DOE cells, but not single‐OE *Pgc‐1α* or *Mipep* cells, exhibited increased expression of *Phospho1* (Fig. 4A). A recent publication documented the binding of C/EBPβ and PPARγ to the *Phospho1* promoter, implying that these transcription factors regulate *Phospho1* expression [20]. Although we found that the expression of *Pparγ* was decreased in both *Pgc‐1α* OE and DOE cells, that of *Cebpb* was unchanged (Fig. 3B,F). Thus, transcriptional activation by C/EBPβ and PPARγ may not fully account for the *Phospho1* upregulation specific to DOE. One possible explanation for this includes mitochondrial retrograde signaling, a pathway of mitochondria‐to‐nucleus communication that modulates gene expression patterns in response to functional changes in mitochondria by activating several signaling pathways from mitochondria to the nucleus [45]. Although *Mipep* single‐OE cells exhibited no remarkable changes in mitochondria‐related phenotypes, an excess of MIPEP is predicted to influence mitochondrial function and activate retrograde signaling, possibly resulting in upregulated *Phospho1* in collaboration with an excess of PGC‐1α in DOE cells. Further investigations are required to determine whether mitochondrial retrograde signaling occurs in *Mipep* OE and DOE cells.

In mouse WAT, we found that CR upregulated the expression of *Phospho1* as well as *Ckb*, a creatin cycle‐related gene that was not affected by DOE *in vitro* (Fig. 4A,B,E,F), suggesting that transcriptional regulation may differ among creatin cycle‐related factors. CR‐induced expression of *Phospho1* was also confirmed in rat WAT (Fig. S2). These results are partially consistent with a prior study showing that CR induced the expression of *Phospho1* but did not affect other creatin cycle‐related genes in WAT of CR mice [46]. Although CR reportedly promotes the differentiation into UCP1^+^ beige adipocytes via enhanced mitochondrial biogenesis, it is well known that CR lowers core body temperature in mammals [28, 47, 48, 49, 50]. A previous study showed that PHOSPHO1 is abundantly expressed in beige fat of *Ucp1*‐deficient mice and is involved in UCP1‐independent energy expenditure [16]. However, *Phospho1* knockout mice were also found to exhibit greater cold tolerance and higher expression of thermogenic genes in BAT [21]. Therefore, while the significance of the seemingly contradictory actions of CR on thermogenesis is unclear, previous findings and our results suggest that these actions may, at least in part, be mediated by PHOSPHO1.

PHOSPHO1 was originally identified as a phosphatase of phospholipids, such as phosphoethanolamine and phosphocholine. To our knowledge, there is no direct evidence of the link between CR and phospholipid metabolism in WAT. However, Ames dwarf mice, which are similar to general CR models in their deficiency of growth hormone and prolonged longevity, reportedly exhibit decreased phospholipids in WAT [51]. In addition, another study revealed aging‐related alterations in plasma membrane lipid composition of adipose tissue [52]. Such findings imply that CR‐induced PHOSPHO1 might contribute to the modulation of the amount and balance of plasma membrane phospholipids, thereby regulating the morphology or function of adipocytes in WAT.

The present study found that DOE of two CR‐induced mitochondrial factors, *Pgc‐1α* and *Mipep*, suppressed white adipocyte differentiation and augmented the levels of *Phospho1* in 3T3‐L1 cells. Unexpectedly, DOE cells did not show significant changes in mitochondrial morphology and function apart from enhanced mitochondrial biogenesis. This might be attributable to the excessive overproduction of PGC‐1α and MIPEP. Indeed, an increase in PGC‐1α expression at a level mimicking that induced by CR has been demonstrated to cause transcriptional and functional metabolic changes [53]. The same could be true for excessive MIPEP production. Hence, further experiments using moderate co‐OE of *Pgc‐1α* and *Mipep* might be expected to lead to mitochondrial activation similar to that observed in CR animals.

In conclusion, the findings of this study provide new insights into the association between CR‐induced mitochondrial factors and regulation of the cellular characteristics of adipocytes. Further research on this topic will enhance our understanding of mitochondria‐related mechanisms of CR.

## Conflict of interest

The authors declare no conflicts of interest.

## Peer review

The peer review history for this article is available at https://www.webofscience.com/api/gateway/wos/peer‐review/10.1002/2211‐5463.70077.

## Author contributions

MI and KT: conceptualization, formal analysis, investigation, visualization, methodology, writing; AH: formal analysis, investigation, validation; KK, YN, YM and TN: investigation, methodology; RM: investigation, resources, methodology; YH: conceptualization, supervision, funding acquisition; MK: conceptualization, resources, formal analysis, supervision, funding acquisition, investigation, visualization, methodology, writing.

## Supporting information


**Fig. S1.** Evaluation of lipid accumulation in double OE (DOE) of *Pgc‐1α* and *Mipep* cells under the condition of inhibited β‐oxidation. Representative images (upper) and quantitation of relative intensity (lower) of oil red O staining 3T3‐L1 cells with mock infection (Mock) or double overexpression (DOE) of *Pgc‐1α* and *Mipep* followed by differentiation into adipocytes for 12 days in the presence or absence of Etomoxir (concentrations: 0, 1, 5 μm) from days 2 to 12; *n* = 3 per group. Scale bars = 100 μm. Values expressed as means ± standard deviation. Differences were statistically analyzed using the Tukey–Kramer test; *P < 0.05, **P < 0.01, ***P < 0.005.
**Fig. S2.** *Phospho1* expression in white adipose tissue (WAT) of rats under caloric restriction (CR). Quantitative real‐time PCR (RT‐PCR) analysis of the mRNA expression levels of *Phospho1* in the white adipose tissue of two feeding groups of rats: *ad libitum* (AL; *n* = 5) and CR (*n* = 6). RT‐PCR data were normalized to *Rps18* expression levels. Values expressed as means ± standard deviation. Differences between values were statistically analyzed using Student's *t*‐test; ***P < 0.001.

## Data Availability

All data are available from the corresponding author upon reasonable request.
