# Supplementary material for: Co‐overexpression of the caloric restriction‐induced mitochondrial factors PGC‐1α and MIPEP upregulates Phospho1 expression in adipocytes
Source: FEBS Open Bio. 2025 Jun 26;15(10):1643–57. doi: 10.1002/2211-5463.70077 (PMC12485865; doi:10.1002/2211-5463.70077)
Supplement: Supplementary file 1 — Fig. S1. Evaluation of lipid accumulation in double OE (DOE) of Pgc‐1α and Mipep cells under the condition of inhibited β‐oxidation. Representative images (upper) and quantitation of relative intensity (lower) of oil red O staining 3T3‐L1 cells with mock infection (Mock) or double overexpression (DOE) of Pgc‐1α and Mipep followed by differentiation into adipocytes for 12 days in the presence or absence of Etomoxir (concentrations: 0, 1, 5 μm) from days 2 to 12; n = 3 per group. Scale bars = 100 μm. Values expressed as means ± standard deviation. Differences were statistically analyzed using the Tukey–Kramer test; *P < 0.05, **P < 0.01, ***P < 0.005. Fig. S2. Phospho1 expression in white adipose tissue (WAT) of rats under caloric restriction (CR). Quantitative real‐time PCR (RT‐PCR) analysis of the mRNA expression levels of Phospho1 in the white adipose tissue of two feeding groups of rats: ad libitum (AL; n = 5) and CR (n = 6). RT‐PCR data were normalized to Rps18 expression levels. Values expressed as means ± standard deviation. Differences between values were statistically analyzed using Student's t‐test; ***P < 0.001. [file FEB4-15-1643-s001.pdf]

Supplementary Figure 1

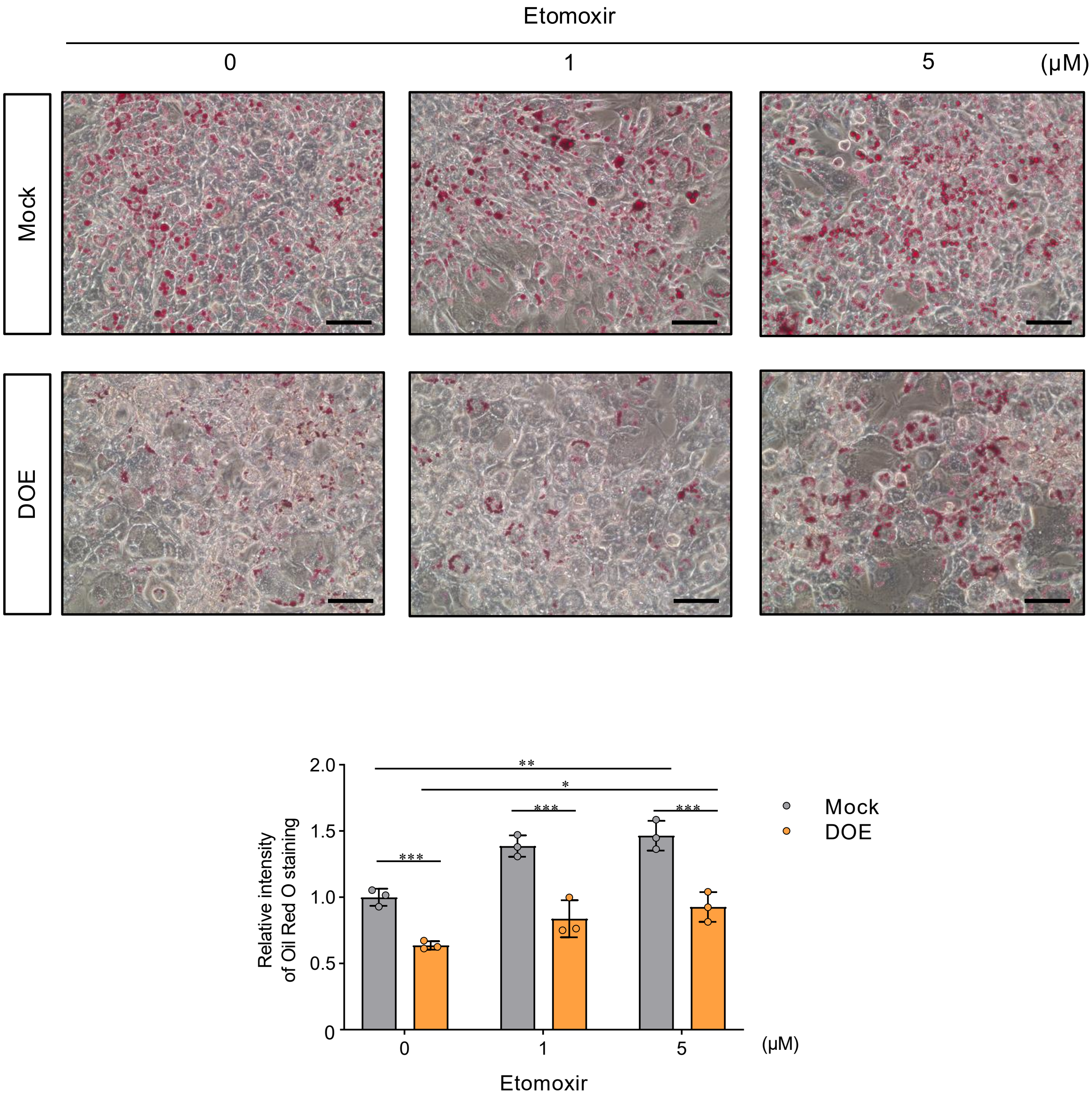

Supplementary Figure 1

Evaluation of lipid accumulation in double OE (DOE) of *Pgc-1α* and *Mipep* cells under the condition of inhibited β-oxidation.

Representative images (upper) and quantitation of relative intensity (lower) of oil red O staining 3T3-L1 cells with mock-infection (Mock) or double overexpression (DOE) of *Pgc-1α* and *Mipep* followed by differentiation into adipocytes for 12 days in the presence or absence of Etomoxir (concentrations: 0, 1, 5 μM) from days 2 to 12; n = 3 per group. Scale bars = 100 μm. Values expressed as means ± standard deviation. Differences were statistically analyzed using the Tukey-Kramer test; $^*P < 0.05$, $^{**}P < 0.01$, $^{***}P < 0.005$.

Supplementary Figure 2

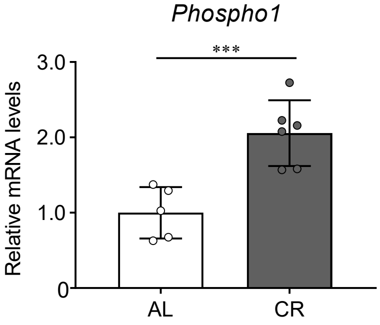

Supplementary Figure 2

***Phospho1* expression in white adipose tissue (WAT) of rats under caloric restriction (CR).** Quantitative real-time PCR (RT-PCR) analysis of the mRNA expression levels of *Phospho1* in the white adipose tissue of two feeding groups of rats: *ad libitum* (AL; n = 5) and CR (n = 6). RT-PCR data were normalized to *Rps18* expression levels. Values expressed as means ± standard deviation. Differences between values were statistically analyzed using Student's t-test; ****P* < 0.001.
